# Endohedral Gd-Containing Fullerenol: Toxicity, Antioxidant Activity, and Regulation of Reactive Oxygen Species in Cellular and Enzymatic Systems

**DOI:** 10.3390/ijms23095152

**Published:** 2022-05-05

**Authors:** Ekaterina S. Sushko, Natalia G. Vnukova, Grigoriy N. Churilov, Nadezhda S. Kudryasheva

**Affiliations:** 1 Institute of Biophysics SB RAS, Federal Research Center “Krasnoyarsk Science Center SB RAS, 660036 Krasnoyarsk, Russia; n-qdr@yandex.ru; 2 Institute of Physics SB RAS, Federal Research Center “Krasnoyarsk Science Center SB RAS, 660036 Krasnoyarsk, Russia; nata_hd@rambler.ru (N.G.V.); churilov@iph.krasn.ru (G.N.C.); 3 Siberian Federal University, 660041 Krasnoyarsk, Russia

**Keywords:** endohedral fullerenol, gadolinium, toxicity, oxidative stress, antioxidant activity, reactive oxygen species, bioluminescence bioassay, hormesis

## Abstract

The Gd-containing metallofullerene derivatives are perspective magnetic resonance imaging contrast agents. We studied the bioeffects of a water-soluble fullerene derivative, gadolinium-endohedral fullerenol, with 40–42 oxygen groups (Gd@Fln). Bioluminescent cellular and enzymatic assays were applied to monitor toxicity and antioxidant activity of Gd@Fln in model solutions; bioluminescence was applied as a signaling physiological parameter. The Gd@Fln inhibited bioluminescence at high concentrations (>2·10^−1^ gL^−1^), revealing lower toxicity as compared to the previously studied fullerenols. Efficient activation of bioluminescence (up to almost 100%) and consumption of reactive oxygen species (ROS) in bacterial suspension were observed under low-concentration exposure to Gd@Fln (10^−3^–2·10^−1^ gL^−1^). Antioxidant capability of Gd@Fln was studied under conditions of model oxidative stress (i.e., solutions of model organic and inorganic oxidizers); antioxidant coefficients of Gd@Fln were determined at different concentrations and times of exposure. Contents of ROS were evaluated and correlations with toxicity/antioxidant coefficients were determined. The bioeffects of Gd@Fln were explained by hydrophobic interactions, electron affinity, and disturbing of ROS balance in the bioluminescence systems. The results contribute to understanding the molecular mechanism of “hormetic” cellular responses. Advantages of the bioluminescence assays to compare bioeffects of fullerenols based on their structural characteristics were demonstrated.

## 1. Introduction

Carbon nano-objects are of great interest for different fields of medicine, pharmacology, and biotechnology due to their specific biological activity [1,2,3]. Fullerenes are carbon nanomaterials known for their unique cage structure. Numerous fullerene-based compounds with different biological targets have been synthesized; biomedical and bioengineering aspects for their application are currently under intensive review [4,5,6,7,8,9,10]. Fullerenes and their derivatives are prospective candidates for anticancer or antimicrobial therapy, cytoprotection, enzyme inhibition, controlled drug delivery, contrast-based or radioactivity-based diagnostic imaging, radio-protection, photosensitization, and biomimetics. Fullerene properties such as antioxidant or pro-oxidant potential, toxicity, membranotropicity, protein-binding affinity, and antiviral, antimicrobial, and anti-apoptotic ability are currently under investigation. It is known [11] that the chemical structure of fullerene derivatives allows them to neutralize reactive oxygen species effectively. This process can occur place in all media: solutions of low-molecular-weight and high-molecular-weight compounds, biomolecules, cells, and tissues.

Fullerenols are water-soluble polyhydroxylated derivatives of fullerenes. Similar to fullerenes, fullerenols are electron-deficient structures and this property makes them efficient catalyzers in biochemical reactions, as well as prospective pharmaceutical drugs. Additionally, fullerenols are amphiphilic structures: fragments of fullerene skeleton provide them with affinity to hydrophobic fragments of enzymes and lipid structures of cellular membranes, while the hydroxyl groups, with aqueous solubility [1,2]. Hydroxyl substituents distort the π-electron system conjugation of the fullerene skeleton, change the electron-acceptor ability of nanoparticles and can therefore affect their catalytic activity. Hence, the variation of the number of the hydroxyl substituents in the fullerenol structures can change the toxicity and antioxidant activity of the fullerenol nanoparticles.

The amphiphilic properties and electron-acceptor ability provide a wide range of biological effects of the fullerenols: from neutralization of free radicals [12] to cell protection and drug transportation [1,12,13,14]. The antioxidant properties endow fullerenols with the ability to neutralize reactive oxygen and nitrogen species [15,16,17,18,19], and to function as radioprotectors [17], antitumor [20], or neurological [12,17,18,19] drugs. The biological activity of C_60_-fullerenols with different number of hydroxyl groups have been intensively studied over the past decades [12,13,14,15,16]. Toxic and antioxidant effects of the fullerenols are revealed.

It is known that gadolinium-based preparations have potential in magnetic resonance imaging and cancer research due to unique paramagnetic properties of gadolinium [21,22]. The most medically used gadolinium-containing compounds are gadodiamide, gadopentetate dimeglumine, gadoterate meglumine, etc. [23,24]; however, there are concerns that these substances may be toxic [24]. They are known to lead to severe skin and systemic diseases (gadolinium ions can exhibit neurological and cardiovascular toxicity [25]), renal dysfunction [26], and intracranial deposition of gadolinium [27]. The problem of toxicity of gadolinium preparations can be solved by involvement of gadolinium into the fullerene carcass. Gd@C_82_ is a common gadolinium metallofullerene (gadofullerene), consisting of a core of a gadolinium (Gd) atom and a closed outer shell of 82 carbon atoms [28,29]. It is supposed [21,30,31,32,33,34,35,36,37,38] that an endohedral fullerene complex and its derivatives can be widely used in biomedicine as novel magnetic resonance imaging (MRI) contrast agents. The high electron affinity of Gd@C_82_ suggests its ionic structure, in which the inner paramagnetic metal ion Gd^3+^ is encapsulated in the negatively charged carbon cage, thus forming a dipole charge-transfer complex Gd^3+^@C_82_^3−^, which prevents a release of toxic ions Gd^3+^ into the bioenvironment [34]. Ionic conjecture for Gd@C_82_ is widely accepted by the scientific community [30].

Endohedral Gd-containing fullerenols, water-soluble derivatives of Gd@C_82_, are good candidates for biomedical applications due to their solubility in water. It is shown that they improve the effectiveness of cancer therapy in combination with chemotherapy [39].

Bioluminescence-based assays are appropriate candidates to study the biological activity of fullerene derivatives due to their simplicity, sensitivity, and high rates of analysis (1–20 min). The bioluminescent assays use the luminescence intensity as a physiological testing parameter; this parameter can easily be measured using simple physical devices. These advantages allow investigators to conduct a large number of tests under comparable conditions during a short time-period; therefore, these tests are adapted to extensive statistical processing, particularly, at low-concentration (low-intensity or low-dose) exposures, which usually produce “noisy” responses and they are described in terms of “stochasticity”.

The bioluminescence bacteria-based assay is commonly used; it has been applied for more than fifty years to monitor the “general” toxicity of complex media [40,41,42,43,44]. The use of the bioluminescence enzymatic assay is a relatively new direction in the toxicology practice [45,46]. As a rule, the enzymatic bioluminescent assay is based on two coupled enzymatic reactions of luminous bacteria (presented in Section 3.2). We used this assay to assess two toxicity types—“general” and “oxidative” ones. The “general” toxicity type integrates all the interactions of the bioluminescent assay system with toxic compounds: redox processes, polar and non-polar binding, etc.; it uses the bioluminescence intensity as a testing parameter. The “oxidative” toxicity type is attributed to the redox properties of toxic compounds only; it uses another testing kinetic parameter—the bioluminescence delay period [47]. The differences in ”general” and “oxidative” toxicity evidence the involvement of the hydrophobic (non-polar) interactions in the toxic effect.

Previously [48,49,50,51], we suggested an original bioluminescence-based method to evaluate antioxidant properties of bioactive compounds. The method involved (1) application of model solutions of oxidizers to produce an “artificial oxidation stress” on luminous bacteria (or their enzyme systems); (2) evaluation of the toxic effect of the model oxidizer solutions on the bioassay systems; (3) exposition of the oxidizer solutions to the bioactive compounds and evaluation of changes in the toxicity (i.e., detoxification of the model oxidizer solutions or, in other words, “antioxidant” effect); (4) calculation of coefficients of antioxidant activity of the bioactive compounds. As we can use the cellular (luminous bacteria) or enzymatic (bacterial enzymes) bioassay systems, we can compare the antioxidant effects at cellular and enzymatic levels. Additionally, differences in the ”general” and “oxidative” toxicity provide information on the amphiphilic properties of the bioactive compounds.

Humic substances, products of natural decomposition of organic matter in soils, coals, and bottom sediments, were the first bioactive compounds that we studied using this approach [49,50,51]. Later, the bioeffects of gold nanoparticles were analyzed in [52], the toxicity and antioxidant activity of a series of different fullerenol nanoparticles were evaluated and compared in [53,54,55,56,57,58,59], prooxidant properties of mignetide nanoparticles were demonstrated [60]. Thus, we have demonstrated that bacteria-based and enzyme-based bioluminescence assays exhibit strong potential as appropriate tools for studying and comparing the bioeffects of nanocompounds of different structures.

The question arises: does the involvement of the gadolinium atom to the fullerene cage change the toxicity and antioxidant activity of fullerenol? Recent theoretical calculations [61] predicted that the Gd atom promotes the chemical reactivity and electrophilic properties of fullerenol cages. It was shown in [6,7,8,9] that electron affinity and average polarizability of Gd@C_82_ are more significant than those for pristine fullerenes [62,63,64,65]; hence, it is a stronger electron donor and acceptor. Therefore, the fullerenol can act an efficient antioxidant in addition to its application as an MRI contrast agent.

Antioxidant properties of bioactive compounds are supposed to be concerned with reactive oxygen species (ROS) in biological systems. The correlations between the ROS content and the toxic/antioxidant effects of bioactive compounds (fullerenols, gold nanoparticles, and radionuclides) in suspensions of luminous marine bacteria were studied in [52,53,59,66,67,68,69]. The role of ROS in the toxic and antioxidant effects of endohedral Gd-containing fullerenols is of high interest; it has not been studied experimentally yet.

In this work, we studied the toxic and antioxidant properties of the endohedral fullerenol Gd@C_82_O_x_(OH)_y_, where x + y = 40–42, which is further referred to as Gd@Fln. The bacteria-based and enzyme-based bioluminescence assays were used to evaluate toxic and antioxidant characteristics of Gd@Fln. The toxic characteristics of Gd@Fln were determined in high-concentration ranges; the low-concentration activation effects of Gd@Fln were found. The bioeffects of Gd@Fln were compared to those of other fullerenols studied earlier. Correlations between the ROS content and the toxic/activating characteristics of Gd@Fln were found in different Gd@Fln concentration ranges. The conditions of model oxidative stress (i.e., solutions of model oxidizers of organic and inorganic types) were applied to evaluate the antioxidant coefficients of Gd@Fln; they were determined at different concentrations and times of exposure to Gd@Fln. Correlations between the antioxidant coefficients and the ROS content were found and discussed. The role of hydrophobic interactions, electron affinity and ROS consumption in the bioeffects of Gd@Fln were taken into consideration. Additionally, in Section 2.2.3 we elucidate the conditions of oxidative stress; the section compares the ROS content in oxidizer solutions in the absence and presence of the biological structures (cells and enzymes).

## 2. Results and Discussion

### 2.1. Effects of Gd@Fln on Bioluminescence and ROS Content

We studied the effects of Gd@Fln of different concentrations (10^−14^–3 gL^−1^) on the bioluminescence of bacterial cells and enzymatic systems.

**Figure 1:**
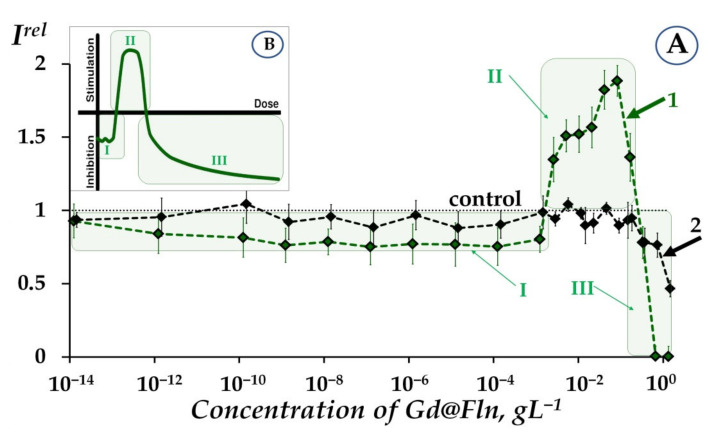
(**A**) Relative bioluminescence intensity, *I^rel^*, at different concentrations of fullerenol Gd@Fln in bacterial suspension (1) and enzymatic system (2). The 5-min exposure. (**B**). Scheme of hormesis dose-effect model is presented according to [67]. Hormetic stages: I—stress recognition, II—physiological activation, III—inhibition of vital functions. “Control” corresponds to the absence of Gd@Fln in the experimental solutions.

Figure 1A presents a dependence of the relative bioluminescent intensity (*I^rel^*, Equation (1), Section 3.2) of luminous bacterial suspensions (curve 1) and enzymatic system (curve 2) on the concentration of Gd@Fln, at initial time of exposure to Gd@Fln (5-min).

It is seen from Figure 1A that dependence of *I^rel^* on fullerenol concentration in bacterial suspension (curve 1) includes three stages: (I) moderate inhibition (*I^rel^* < 1) at 10^−14^–10^−3^ gL^−1^, (II) activation (*I^rel^* > 1) at 10^−3^–2·10^−1^ gL^−1^, and (III) inhibition (*I^rel^* < 1) at 2·10^−1^–3 gL^−1^.

It should be noted that there exists a difference between bioluminescence kinetics under exposure to higher and lower Gd@Fln concentrations. Figure S1 (Supplementary Materials) presents examples of these kinetics. The conventional border between higher and lower concentration ranges was ca. 2·10^−1^ gL^−1^, it was taken into consideration during the course of further data analysis. Studies of higher- and lower-concentration effects of Gd@Fln are presented in Section 2.1.1 and Section 2.1.2, respectively.

#### 2.1.1. Toxicity of Gd@Fln via Bioluminescence Enzymatic and Cellular Assays at High-Concentration Ranges

We examined the toxicity factor of fullerenol Gd@Fln using cellular and enzymatic bioluminescence assays. As is evident from Figure 1A, Gd@Fln suppresses bioluminescence of both bacterial and enzymatic systems at high concentrations (>2∙10^−1^ gL^−1^). The suppression is evidence of the fullerenol toxic effect; it is supposed to be concerned with complex multiple processes which resulted in inhibition of membrane and intracellular processes (for bacterial cells) [47,49] or chemical and biochemical reactions (for enzymatic system) by low-molecular and nano- compounds as previously discussed [47,49,54,56,70]. Note, that the inhibition processes are not concerned with the peculiarities of the luminescence registration, since “concentration quenching” resulting from collisional intermolecular interactions was initially excluded (See Section 3.2). The values of *EC*_50_ for Gd@Fln were determined as 0.46 and 1.4 gL^−1^ for the bacterial suspension and enzymatic system, respectively. It is evident that the bacterial system revealed higher sensitivity to Gd@Fln (i.e., lower value of *EC*_50_), likely due to hydrophobic interactions with cellular membrane involvement. Similar results were observed earlier with other fullerenols of different structures [53] (fullerenol with exohedral iron atom was excluded due to specific action of iron on metabolism of the bacterial cells). The *EC*_50_ values of fullerenols of different structures were determined earlier under similar conditions; they ranged from 0.003 to 0.031 gL^−1^ for the bacterial suspension [53] and from 0.002 to 0.092 gL^−1^ for the enzymatic system [53,59]. Hence, toxicity of Gd@Fln is lower (i.e., *EC*_50_ values are higher in both bioluminescent systems) than that of the other fullerenols studied earlier [53,59]. This effect can be explained by larger cage size of Gd@Fln (involving 82 carbon atoms) and its tendency towards aggregation. The aggregate formation was studied in detail in [21,71,72,73] with the example of endohedral fuller enol with 22 hydroxyl groups, Gd@C_82_(OH)_22_; polyanion nano-aggregation into cluster in aqueous solutions was demonstrated. The aggregation might prevent intensive interactions of Gd@Fln with cellular membranes or water-soluble enzymes.

#### 2.1.2. Low-Concentration Effects of Gd@Fln

Bioluminescence activation of bacteria (*I^rel^* > 1, Figure 1A, curve 1) was found at low-concentration exposure to Gd@Fln (10^−3^–2·10^−1^ gL^−1^). The activation was significant—up to almost 100%, as compared to control. The bacterial response to Gd@Fln corresponds to the conventional “hormesis” model [74,75,76,77], which is presented in Figure 1B. It is known that the model includes, in the broadest case, three stages of the biological dose-dependent response—stress recognition (I), activation (II), and inhibition of organismal functions or toxic effect (III). As a concept, hormesis involves favorable biological responses to low exposures of stressors [78,79].

In contrast to bacteria, enzymatic response to Gd@Fln did not show bioluminescence activation (curve 2, Figure 1A). This is an indication that the bacterial activation (curve 1, Figure 1A) is concerned with indirect effects on bioluminescent reaction and probably related to cell membrane processes with hydrophobic interactions involved.

Previously [53,54,55,56,57,58,59], we did not observe low-concentration activation of bacterial bioluminescence by the other fullerenols; only high-concentration inhibition (toxic effect) was found. This difference is likely evidence of higher reactivity and reversible electron-acceptance ability of Gd@Fln [65,80,81]. Previous experimental and theoretical results support this supposition. It was found in [82], that Gd endofullerene is characterized by a significantly (one-and-a-half to two orders of magnitude) higher reactivity with respect to C_60_ and C_70_, which can be accounted for by the nonuniform distribution of electron density of the fullerene cage due to the presence of the endohedral atom. The electron affinity of Gd@C_82_ is more significant than those for pristine C_60_ and C_70_ (1.25 and 1.19 times, respectively); the insertion of Gd into a C_82_ cage increases the electron affinity to 3.3 eV [64]. Gd^3+^@C_82_^3−^ can be involved in free-radical addition reactions, which can change the electronic structure of the inner cluster and affect its configuration [83].

#### 2.1.3. Involvement of ROS in the Responses of Bacterial and Enzymatic Systems to Gd@Fln

It should be noted that we initially studied time-courses of ROS content in control samples (i.e., without Gd@Fln) of bacterial and enzymatic systems for the time of bioluminescent experiment, 45 min. We found an increase in ROS content (from 1.9·10^−5^ M to 4.7·10^−5^ M) in the control enzyme solutions, while the ROS content in the control bacterial suspensions was almost constant—about 4.5·10^−6^ M. The explanation is likely the following: the increase mentioned can be explained with dark processes associated with the accumulation of peroxide compounds in the reaction of bacterial luciferase [84]. Bacterial cells are likely able to balance ROS content and maintain homeostatic levels of ROS involved in metabolic coupled redox reactions.

In order to verify the role of ROS in the bioeffects of Gd@Fln (Figure 1A), we determined ROS content in bacterial suspensions and enzymatic systems. Dependences of ROS content on time of exposure to fullerenol Gd@Fln were studied at different concentrations of Gd@Fln solutions (10^−13^–3 gL^−1^). Examples of kinetics of relative ROS content, *ROS^rel^*, at two concentrations of fullerenol Gd@Fln are presented in Figure S2 (Supplementary Materials).

**Figure 2:**
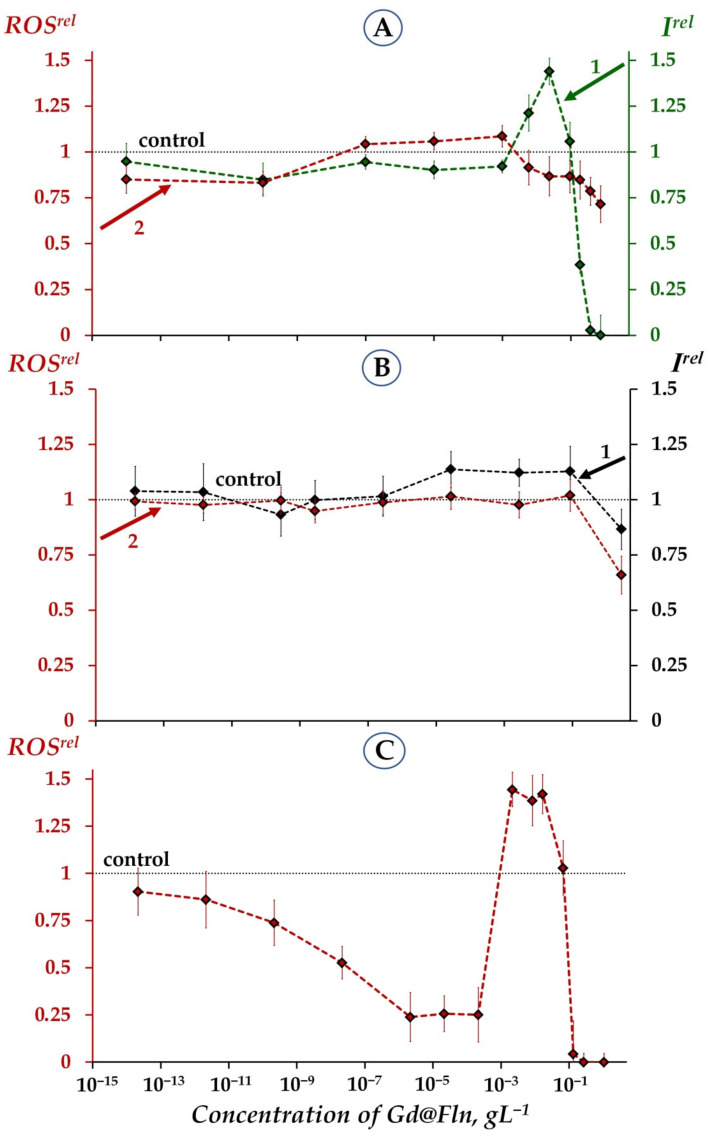
Relative bioluminescence intensity, *I^rel^*, (1) and relative ROS content, *ROS^rel^*, (2) in bacterial suspension (**A**), enzymatic system (**B**) and distilled water (**C**) at different concentrations of fullerenol Gd@Fln. Time of exposure to Gd@Fln was 1 min. Concentration of ROS in the control bacterial suspension was ~4.5·10^−6^ M, in the control enzymatic sample—1.9·10^−5^ M, in distilled water—3·10^−7^ M. “Control” corresponds to the absence of Gd@Fln in the experimental solutions.

Values of *ROS^rel^* were determined along with *I^rel^* in bioluminescence experiments and presented in Figure 2 for bacterial (Figure 2A) and enzymatic (Figure 2B) systems.

We analyzed correlations between concentration dependencies of *I^rel^* and *ROS^rel^* for bacterial suspensions (Figure 2A) in a low-concentration range of Gd@Fln: 10^−7^–10^−1^ gL^−1^. This range revealed a negative correlation (r = −0.8, *p* < 0.05) and therefore demonstrated the inverse dependence between bioluminescence intensity and ROS content. We can conclude that the bacterial bioluminescence activation by Gd@Fln (*I^rel^* > 1, curve 1, Figure 2A) is related to the moderate decrease in ROS (*ROS^rel^* < 1, curve 2, Figure 2A), probably as a result of intensification of ROS consumption by the bacteria induced by fullerenol [84,85]. This conclusion infers the molecular mechanism of “hormetic” response of the bacterial cells to fullerenol. A higher concentration range of Gd@Fln (10^−1^–8·10^−1^ gL^−1^) revealed a positive correlation between concentration dependences of *I^rel^* and ROS content (r = 0.8, *p* < 0.05). This result reveals different molecular mechanisms of Gd@Fln influence on bacteria at lower-concentration and higher-concentration ranges, resulting in bioluminescence activation and inhibition, respectively. Inhibition and activation of bacterial bioluminescence intensity by ROS was reported previously for bacterial and enzymatic assays, hydrogen peroxide was applied by the authors as a representative of ROS [86,87].

Figure 2B presents the dependences of *I^rel^* and *ROS^rel^* on concentrations of Gd@Fln in the enzymatic system (curves 1 and 2, respectively). No reliable bioluminescence activation was observed in the enzyme solutions (curve 1, Figure 2B), similar to the previous results of the analogous experiment presented in Figure 1A, curve 2. A positive correlation (r = 0.9, *p* < 0.05, 10^−7^–3 gL^−1^) between the concentration dependences of *I^rel^* and *ROS^rel^* was found, Figure 2B.

It should be noted that a similar high-concentration decline in both of *I^rel^* and *ROS^rel^* as well as positive correlation between these parameters were reported earlier for enzymatic system exposed to the other fullerenol (C_60_) with low number of oxygen substituents [59]. This correlation was suggested to have resulted from the consumption of ROS in the course of the bioluminescence reaction. The physicochemical mechanism of fullerenol’s influence on the enzymatic assay system is likely due to its ability to neutralize free radicals [53] including peroxide radicals. It is known that one of the intermediates of the bioluminescent luciferase reaction (reaction 2, Section 3.2), flavin peroxy-semiacetal [88,89], is a peroxide that is categorized as a ROS. Hence, the decrease in ROS content (*ROS^rel^* < 1), at high fullerenol concentrations can account for the inhibition of the bioluminescent reaction (reaction 2, Section 3.2). The bacterial bioluminescence reaction can be considered as a model of enzymatic oxygen-dependent reactions taking place in all living organisms.

Hence, intermediate conclusions from the results in Figure 2A,B are the following:

1. Similar to the previous results [53,59], the toxic effects of Gd@Fln can be concerned with the lack of ROS (*ROS^rel^* < 1) in bacteria-based and enzyme-based assay systems. It takes place at high fullerenol concentrations (>2∙10^−1^ gL^−1^, Figure 1A).
2. Additionally, a moderate ROS decay (*ROS^rel^* < 1) at low-concentration fullerenol exposure (10^−3^ gL^−1^–2∙10^−1^ gL^−1^) might be related to the activation of bacterial bioluminescence as a result of ROS consumption.

As previously mentioned, it is commonly recognized that only the excess of ROS leads to toxic effects which resulted in DNA damage and cell death [90,91,92]. Our results develop our understanding of ROS functions in biological systems revealing complex interrelations between ROS content and physiological efficiency. Probably, there exists an optimum range of ROS concentrations, which is balanced naturally by living systems.

Figure 2C presents ROS content in aqueous solutions of Gd@Fln. The complexity of the concentration dependence is evident from this Figure. A low-concentration range (<10^−4^ gL^−1^) shows a decline of ROS content as compared to control (*ROS^rel^* < 1); hence, this range alone provides antiradical activity of fullerenol. A higher concentration range (10^−4^–10^−1^ gL^−1^) demonstrates an increase in ROS content (*ROS^rel^* > 1). Previously, we did not observe such a district increase in ROS content in aqueous solutions of other fullerenols [53]; mechanism of this phenomenon should be further elucidated. However, we can preliminarily suggest that the decay in ROS-neutralizing ability might be concerned with dipole nature of Gd@Fln and formation of aggregates. The high efficiency of aggregate formation was confirmed previously: it was found that clusters of endohedral metal-fullerenes reach hundreds of nanometers [93,94], in contrast to tens–nanometer clusters of empty fullerens [95].

Nevertheless, it is seen that the discussed concentration range with high ROS content (10^−4^–10^−1^ gL^−1^, Figure 2C) provides the bioluminescence activation, noticeable or slight for bacteria (Figure 1 and Figure 2A curves 1) or enzymes (Figure 2B, curve 1), respectively, with *ROS^rel^*-values closed to control (Figure 2A,B, curves 2). The supposition can be made that biological structures, cellular or enzymatic, mitigate deviations of ROS content in environment via intensification of the bioluminescence function. In previous works, the detoxification of reactive oxygen by luciferase reaction was discussed in [96]; in classic work by Wilson and Hastings [97], authors stated that luciferase “transforms excess energy… into light energy instead of being all lost as heat”.

### 2.2. Antioxidant Activity of Fullerenol and ROS Content

To study antioxidant activity of fullerenol Gd@Fln, we excluded a high-concentration range of Gd@Fln inhibiting bioluminescence (>2∙10^−1^ gL^−1^ for both bacterial and enzymatic systems) based on the results presented in Section 2.1.1, Figure 1A.

Antioxidant activity of fullerenol Gd@Fln was studied under conditions of model oxidative stress, i.e., in model solutions of oxidizers of organic and inorganic types (1,4-benzoquinone and potassium ferricyanide K_3_[Fe(CN)_6_], respectively). We “fixed” conditions of model oxidative stress by using the effective concentrations of oxidizers, *EC*_50_; values of *EC*_50_ are presented in Section 3.2. Bioluminescence intensity of the bacterial and enzymatic systems was measured in the absence and presence of Gd@Fln under conditions of the model oxidative stress; concentrations of Gd@Fln were varied. Antioxidant coefficients of *I^rel^_Ox_* and *T^rel^_Ox_* were calculated and compared to ROS content. Values of *I^rel^_Ox_* > 1 and *T^rel^_Ox_* > 1 were evidence of antioxidant activity of Gd@Fln.

#### 2.2.1. Antioxidant Coefficients I^rel^_Ox_ and ROS Content

Antioxidant coefficients I^rel^_Ox_ (Equation (3), Section 3.2) and relative ROS content, ROS^rel^_Ox_, were calculated in a wide range of Gd@Fln concentrations (10^−14^–2·10^−1^ gL^−1^). Dependences of I^rel^_Ox_ and ROS^rel^_Ox_ on concentration of Gd@Fln were compared.

It should be noted that initially we studied time-courses of ROS content in control samples (i.e., without Gd@Fln) of bacterial and enzymatic systems in model solutions for the time of bioluminescent experiment, 45 min. We found that the ROS content in the control enzyme solutions (enzymes + 1,4-benzoquinone at *EC*_50_ = 10^−5^ M) and in the control bacterial suspensions (bacteria + 1,4-benzoquinone at *EC*_50_ = 8·10^−7^ M) were almost constant—about 4.5·10^−5^ M and 5.8·10^−6^M, respectively.

**Figure 3:**
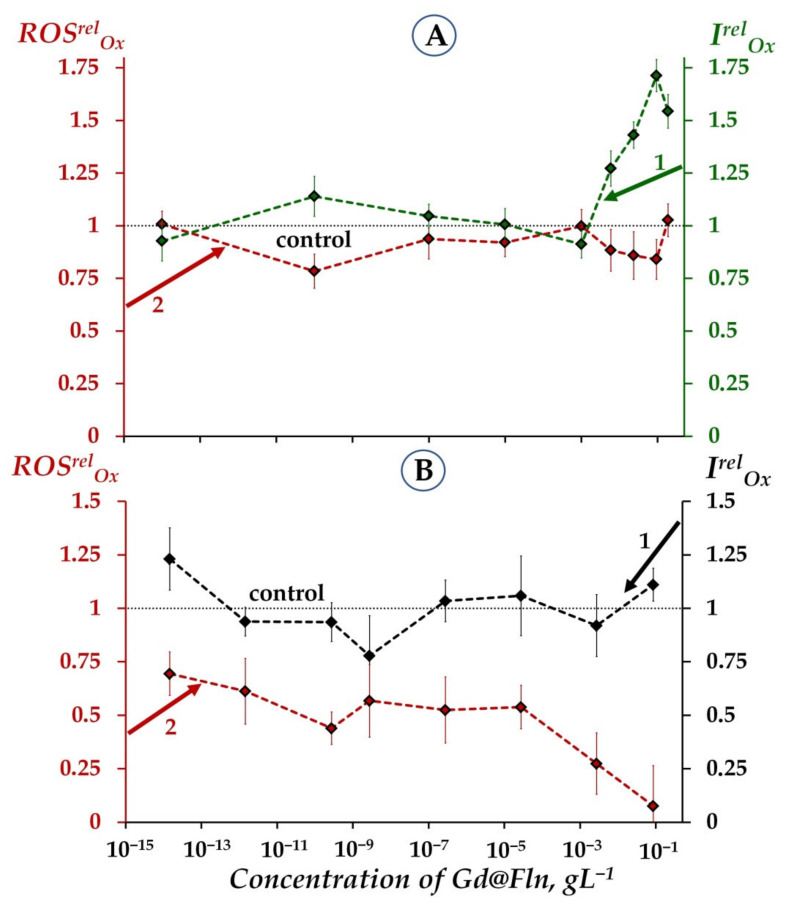
Antioxidant coefficients, *I^rel^_Ox_*, (1) and relative ROS content, *ROS^rel^*, (2) in bacterial suspension (**A**) and enzymatic system (**B**) vs. concentration of fullerenol Gd@Fln. Time of exposure to Gd@Fln was 45 min. Concentrations of ROS in the control bacterial suspension (bacteria + 1,4-benzoquinone at *EC*_50_ = 8·10^−7^ M) and control enzymatic system (enzymes + 1,4-benzoquinone at *EC*_50_ = 10^−5^ M) were 5.8·10^−6^ M and 4.9·10^−5^ M, respectively. “Control” corresponds to the absence of Gd@Fln in the experimental solutions.

Figure 3 presents values of I^rel^_Ox_ and ROS^rel^_Ox_ in solutions of organic oxidizer, 1,4-benzoquinone, in the bacterial and enzymatic systems (Figure 3A,B, respectively).

Figure 3A shows that Gd@Fln detoxifies the 1,4-benzoquinone solutions in bacterial suspension (curve 1) in the concentration ranges of 10^–3^–2·10^−1^ gL^−1^ (I^rel^_Ox_ > 1, *p* < 0.05) with the maximal value of I^rel^_Ox_ = 1.75.

Mitigation of the bacterial response to the oxidative load was observed not only in the solutions of organic oxidizer. In the solutions of inorganic oxidizer, potassium ferricyanide (curve 1, Figure S3, Supplementary Materials), Gd@Fln revealed moderate but reliable deviations of I^rel^_Ox_ from the control (*p* < 0.05) with the maximal value of I^rel^_Ox_ = 1.2 at a concentration range 10^–7^–10^−2^ gL^−1^ (*p* < 0.05).

Similar behavior of other fullerenols was observed earlier [54,55,56,58,59]; higher antioxidant effects were observed in solutions of the organic oxidizer (1,4-benzoquinone). The difference in detoxifying ability of the fullerenols can be related to the hydrophobic/hydrophilic properties of the model oxidizers, and the involvement of hydrophobic fragments of the organic oxidizer in the detoxifying process in cellular or enzymatic systems. Hence, our results can be considered as an additional indirect confirmation of the importance of hydrophobic interactions in the bioeffects of Gd@Fln discussed in Section 2.1.1.

Figure 3A (curve 2) demonstrates that Gd@Fln did not noticeably change ROS content in bacterial suspension + 1,4-benzoquinone at all Gd@Fln concentrations used.

We analyzed correlations between concentration dependences of *I^rel^_Ox_* and *ROS^rel^_Ox_* in bacterial suspensions (Figure 3A) under conditions of oxidative exposure (i.e., in solutions of 1,4-benzoquinone) at a concentration range of Gd@Fln: 10^−14^–2·10^−1^ gL^−1^. This range revealed a moderate negative correlation (r = −0.7, *p* < 0.05, Figure 3A). This correlation demonstrates the inverse dependence between bacterial bioluminescence intensity and ROS content under conditions of oxidative stress, similar to the conditions without redox stress discussed previously (Section 2.1.3, Figure 2A). We can conclude that the mitigation of model oxidative stress in bacterial suspension (i.e., bioluminescence activation) is concerned with the intensification of ROS consumption by the bacteria.

Notably, the maximal antioxidant coefficients of Gd@Fln, *I^rel^_Ox_* in the bacterial suspension rise with increased exposure time: from 1.4 (at 5-min exposure, Figure S4A, Supplementary Materials, curve 1) to 1.75 (45-min exposure, Figure 3A, curve 1), but ROS content does not change throughout the durations of the experiment.

Figure 3B reveals the absence of a noticeable antioxidant effect of Gd@Fln on the bioluminescence intensity of the enzyme system (curve 1): the value of I^rel^_Ox_ was close to 1 in benzoquinone solution. Similarly, Gd@Fln did not affect the enzymatic bioluminescence (I^rel^_Ox_ ≈ 1) in solution of inorganic oxidizer, potassium ferricyanide (curve 2, Figure S3, Supplementary Materials).

The difference in responses of cellular and enzymatic systems to Gd@Fln in oxidizer solutions can be considered as an additional indirect confirmation of the importance of hydrophobic interactions and the involvement of cellular membrane in the bioeffects of Gd@Fln discussed in Section 2.1.1.

In contrast to the bacterial system (Figure 3A, curve 2), the enzymatic system demonstrated about 50% decrease in ROS content at a wide low-concentration range of Gd@Fln (10^−14^–10^−4^ gL^−1^), Figure 3B, curve 2. We can suggest that Gd@Fln of low concentrations entirely neutralized the benzoquinone-induced excess of ROS in the enzyme solution. However, no reliable correlations between I^rel^_Ox_ and ROS^rel^_Ox_ were found in the enzymatic system. The result highlights the complexity of the processes responsible for the antioxidant effect of Gd@Fln.

#### 2.2.2. Antioxidant Coefficients T^rel^_Ox_ and ROS Content

Bioluminescent enzymatic system allows monitoring of not only bioluminescence intensity (I), but also of the bioluminescence induction period (T), Figure 6b, Section 3.2. The first parameter is used to study ‘general’ toxicity of foreign compounds, but the latter parameter is specific to oxidizers and responsible for ‘oxidative’ toxicity [54,98]. It is supposed that ‘oxidative’ toxicity is a function of redox activity of toxic media only, while ‘general’ toxicity is based on complex processes involving redox and polar/apolar interactions in the enzyme system [49].

In order to monitor changes in oxidative toxicity, the T-values were determined at different concentrations of Gd@Fln. The values of T^rel^_Ox_ were calculated according to Equation (4) (Section 3.2).

Figure S5 (Supplementary Materials) demonstrates the dependences of T^rel^_Ox_ on the concentration of fullerenol Gd@Fln in solutions of 1,4-benzoquinone (curve 1) and K_3_[Fe(CN)_6_] (curve 2). Antioxidant effects (T^rel^_Ox_ > 1) were found in the solutions of both oxidizers; however, the average values of T^rel^_Ox_ were low and did not exceed 1.1 in both cases.

Hence, we found that the antioxidant coefficients T^rel^_Ox_ of Gd@Fln, calculated using induction bioluminescence period were lower than antioxidant coefficients I^rel^_Ox_, calculated using bioluminescence intensity. Similar observations were made in our previous studies of the other fullerenols [59]. This result provides more evidence of the importance of hydrophobic interactions in antioxidant activity of Gd@Fln.

#### 2.2.3. Modeling of Oxidative Stress Conditions through ROS Content in Oxidizer Solutions

Modeling of conditions of oxidative stress is a subject of special interest; the content of ROS in solutions of model oxidizers in the presence and absence of biological molecules or living cells remains unexplored. The solution to this problem is important as it forms a basis for understanding the mechanism of ROS function in organisms and their environments. We try to elucidate this subject using aqueous media of different complexity: (1) solutions of oxidizers, (2) oxidizers + bacterial suspension, and (3) oxidizers + enzyme reactions.

Oxidizers of organic or inorganic types (1,4-benzoquinone or potassium ferricyanide K_3_[Fe(CN)_6_], respectively) were used [70,98,99]. Standard redox potentials of these oxidizers are high: 0.71 V and 0.36 V, respectively [48,49,50,51,98]. Quinone and iron(III) are important representatives of intra-cellular and extra-cellular oxidizers. Additionally, quinones are shown to bind tightly to bacterial enzymes [70]. Quinones are produced environmentally as a result of the oxidative transformation of phenols and occupy the third position in the list of top widespread pollutants (after oil products and metal salts) [100]. Phenolic substances are also synthesized by soil bacteria as molecular signaling molecules in microbial communication and as adaptogens [101] and induce redox transformations in soils and aquifers, especially at low pH in the presence of iron(III) [102,103].

**Figure 4:**
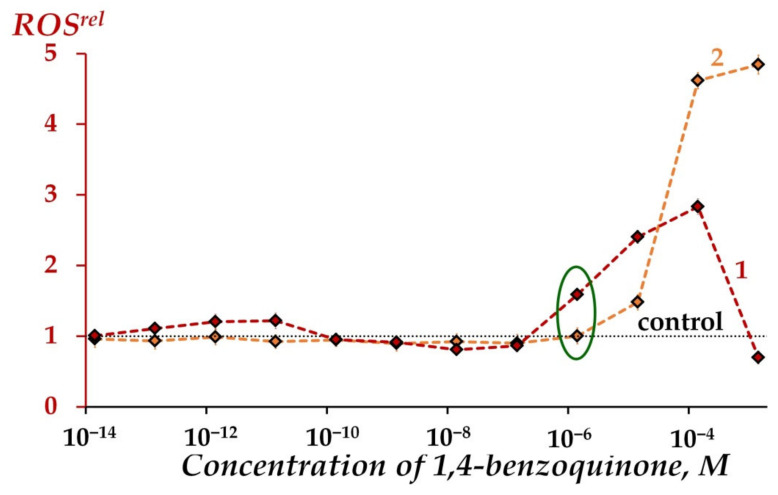
Relative ROS content, *ROS^rel^*, in bacterial suspension (1), physiological 3%NaCl solution without bacteria (2) at different concentrations of 1,4-benzoquinone, 5 min exposure. Concentrations of ROS were 1.3·10^−5^ M and 4.8·10^−6^ M in the control physiological 3%NaCl solution and control bacterial suspension, respectively. Relative ROS content at *EC*_50_ marked with green ellipse. “Control” corresponds to the absence of 1,4-benzoquinone in the experimental solutions.

Figure 4 shows an increase in ROS content in benzoquinone solutions at concentrations > 10^−7^ M for both cases—in iso-osmotic 3%NaCl solutions in the presence and absence of the bacteria (*ROS^rel^ >* 1, curves 1 and 2). It is seen that bacteria mitigate ROS increase at concentration > 10^−5^ M; however, natural bacterial ROS production is effective at low concentrations of 1,4-benzoquinone (10^−7^–10^−4^M) (compare curves 1 and 2 in Figure 4). Hence, the involvement of bacteria in ROS regulation in solutions of organic oxidizer is evident; bacteria increase (at low oxidizer concentrations) or decrease (at higher oxidizer concentrations) ROS content in oxidizer solutions.

**Figure 5:**
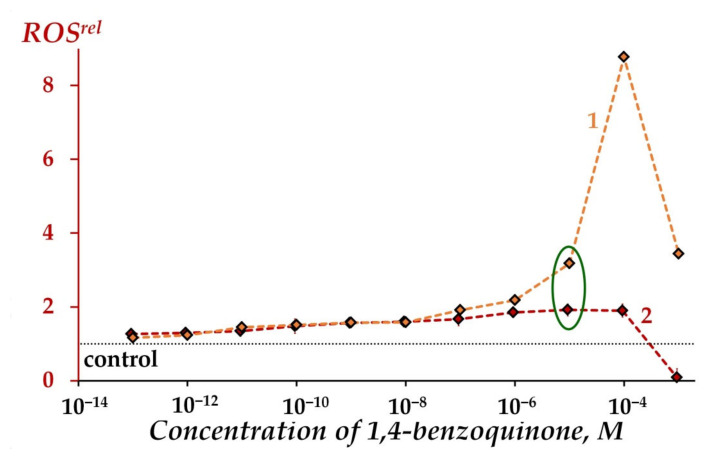
Relative ROS content, *ROS^rel^*, in distilled water (1), enzymatic system (2) at different concentrations of 1,4-benzoquinone, 5 min exposure. Concentrations of ROS were 4.5·10^−7^ M and 1.9·10^−5^ M in distilled water and enzymatic system, respectively. “Control” corresponds to the absence of 1,4-benzoquinone in the experimental solutions.

We found that 1,4-benzoquinone increased ROS content, *ROS^rel^* > 1, in aqueous solutions at all concentrations studied, and in enzyme systems at ≤ 10^−4^ M, curves 1 and 2, Figure 5. This figure demonstrates the mitigation of ROS increase in enzymatic processes (as compared to aqueous solutions) in the entire range of 1,4-benzoquinone concentrations. This effect is a result of the consumption of ROS during the course of oxidative bioluminescence reactions of bacterial luciferase (reaction 2, Section 3.2) as discussed above (See Section 2.1.3).

The differences in effects of bacterial and enzyme reactions on ROS content in aqueous solutions might be concerned with the different level of organization of these two biological systems. This difference is a highly important and interesting subject; it should be clarified in detail during further investigations.

## 3. Materials and Methods

### 3.1. Preparation of Fullerenol Gd@Fln

Gd-endohedral fullerenol Gd@C_82_O_y_(OH)_x_, where x + y = 40–42 (Gd@Fln) was produced by fullerene Gd@C_82_ hydroxylation in nitric acid followed by the hydrolysis of the polynitrofullerenes [104,105,106,107]. Mixture of fullerenes, involving Gd@C_82_, was preliminarily synthesized by carbon helium high-frequency arc plasma at 98 kPa [107,108]. To determine Gd-content, fullerene mixtures were analyzed by atomic emission spectroscopy using calibration curve of the emission intensity versus Gd concentration [109]. The mass spectrum showed encapsulated Gd only (Gd@C_82_). The Gd@C_82_-fullerene content in fullerene mixture was determined as 4.8%. The reaction of complexation with Lewis acids (TiCl_4_) was used for enrichment of the extract of fullerene mixture by endohedral metallofullerenes (Gd@C_82_) [110]. Then, Gd@C_82_ was extracted with carbon disulfide from carbon soot.

The fullerene preparation was characterized with infrared spectroscopy in the KBr matrix using Fourier spectrometer VERTEX 70 (Bruker, Germany). The number of -OH groups was estimated by X-ray photoelectron spectroscopy (XPS) using UNI-SPECS spectrometer (SPECS Gmbh, Germany) [111,112]. Both XPS and infrared (IR) spectra of endohedral Gd-containing fullerenol are presented in Figures S6 and S7 (Supplementary Materials).

### 3.2. Bioluminescence Assay Systems and Experimental Data Processing

Antioxidant activity and toxicity of fullerenol Gd@Fln were evaluated using bioluminescence assay systems, cellular and enzymatic: (1) bacterial assay, i.e., intact marine luminous bacteria *Photobacterium phosphoreum*, strain 1883 IBSO from the Collection of Luminous Bacteria CCIBSO 863, Institute of Biophysics SB RAS, and (2) enzymatic assay, i.e., enzymatic preparation based on the system of coupled enzyme reactions catalyzed by NADH:FMN-oxidoreductase from *Vibrio fischeri* (0.15 a.u.) and luciferase from *Photobacterium leiognathi*, 0.5 mg/mL [113]. The enzyme preparation was produced at the Institute of Biophysics SB RAS (Krasnoyarsk, Russia). Antioxidant activity of Gd@Fln was assessed in model oxidizer solutions (in aqueous or 3% NaCl solutions of K_3_[Fe(CN)_6_] for enzymatic and bacterial systems, respectively, and in 0.05 M phosphate buffer or 3% NaCl solutions of 1,4-benzoquinone for enzymatic and bacterial systems, respectively).

The chemicals were: FMN and tetradecanal from SERVA, Heidelberg, Germany; NADH from ICN Biochemicals, Costa-Mesa, CA, USA; sodium chloride (NaCl) from Khimreactiv, Nizhny Novgorod, Russia; potassium ferricyanide (K_3_[Fe(CN)_6_]) and 1,4-benzoquinone from Sigma-Aldrich, St. Louis, MO, USA; potassium di-hydrogen phosphate (KH_2_PO_4_) and di-potassium hydrogen phosphate (K_2_HPO_4_) from Panreac, Barcelona, Spain. The reagents were of chemical or analytical grade.

To prepare the enzymatic assay system we used 0.1 mg/mL of enzyme preparation, 4∙10^−4^ M NADH, 5.4∙10^−4^ M FMN, and 0.0025% tetradecanal solutions. The NADH and tetradecanal were dissolved in 0.05 M phosphate buffer, pH 6.8, at 25 °C; FMN in distilled water. Concentration of NADH, FMN, and tetradecanal solutions in experimental samples were 1.6∙10^−4^ M, 5.4∙10^−5^ M, 0.00025%, respectively.

The enzymatic assay system is based on the following coupled enzymatic reactions:

(reaction 1) $$NADH+FMN\overset{NADH:FMN-oxidoreduc\;tase}{\to }FMN\cdot H^{-}+NAD^{+}$$

(reaction 2) $$FMN\cdot H^{-}+RCHO+O_{2}\overset{luciferase}{\to }FMN+RCOO^{-}+H_{2}O+h\nu$$

For the cultivation of *P. phosphoreum* 1883 IBSO, the semisynthetic medium containing: 10 gL^−1^ tryptone, 28.5 gL^−1^ NaCl, 4.5 gL^−1^ MgCl_2_·6H_2_O, 0.5 gL^−1^ CaCl_2_, 0.5 gL^−1^ KCl, 3 gL^−1^ yeast extract, and 12.5 gL^−1^ agar was used. *P. phosphoreum* was plated on 25 mL of semisynthetic medium and incubated at 25 °C for a period of 24 h (stationary growth phase corresponding to maximum bioluminescence) in an incubator (WIS-20R, WiseCube Laboratory Instruments, Wertheim, Germany). Prior to experiments, bacteria were collected by pipetting of 3% NaCl solution directly onto the agar to release bacteria. The 3% NaCl solutions were used to imitate a marine environment for the bacterial cells and to balance osmotic processes. The bacterial suspension was diluted to Abs_660_ = 0.025 and stored at 4 °C for 30 min to allow bioluminescence stabilization. The reagents for bacterial cultivation were: tryptone and yeast extract from Dia-M, Moscow, Russia; sodium chloride (NaCl) from Khimreactiv, Nizhny Novgorod, Russia; magnesium chloride hexahydrate (MgCl_2_ 6H_2_O), calcium chloride (CaCl_2_), and potassium chloride (KCl) from Pancreac AppliChem GmbH, Darmstadt, Germany; agar from Difco Laboratories, Detroit, MI, USA.

Toxic effects of Gd@Fln on bioluminescence of bacterial and enzymatic assay systems were characterized by relative bioluminescence intensity, *I^rel^*:

*I^rel^* = *I_F_/I_contr_* (1)

where, *I_contr_* and *I_F_* are maximal bioluminescence intensities in the absence and presence of Gd@Fln, respectively.

The effective concentration of Gd@Fln inhibiting bioluminescence intensity by 50% (*I^rel^* = 0.5), *EC*_50_, were determined to evaluate its toxic effect.

It should be noted that we excluded an additional reason for the bioluminescence suppression—the effect of “optic filter” which is a result of bioluminescence absorption/reabsorption. All experiments with ’colored’ solutions of Gd@Fln excluded effect of ‘optic filter’ (optical density of fullerenol solutions was <0.1 at the maximal bioluminescence light emittance wavelength—490 nm) [114], and this effect did not skew the results of the toxicological measurements.

**Figure 6:**
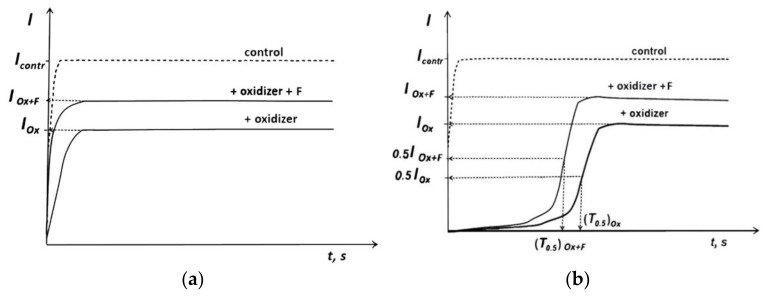
Bioluminescence kinetics in a solution of model oxidizer (Ox) and fullerenol (F): (**a**) cellular assay; (**b**) enzymatic assay.

To study antioxidant properties of Gd@Fln, we used conditions of a model oxidative stress for the bioluminescence assay systems using model oxidizers (*Ox*)—potassium ferricyanide (K_3_[Fe(CN)_6_) and 1,4-benzoquinone; *I_contr_* and *I_Ox_* were measured as shown in Figure 6. Effective concentration *EC*_50_ of the model oxidizers inhibiting bioluminescence intensity by 50%, (*I^rel^_Ox_* = 0.5), *EC*_50_, were determined with bacterial and enzymatic bioluminescence assays:

*I^rel^_Ox_* = *I_Ox_/I_contr_* (2)

where, *I_contr_* and *I_Ox_* are maximal bioluminescence intensities in the absence and presence of model oxidizer, respectively, Figure 6.

The *EC*_50_ values of 1,4-benzoquinone were 8∙10^−7^ M and 10^−5^ M, *EC*_50_ values of K_3_[Fe(CN)_6_] were 10^−3^ M and 10^−6^ M for bacterial and enzymatic assays, respectively. The values are close to those determined earlier [48,50]. The effect of “optic filter” was also excluded in these measurements.

Antioxidant activity of Gd@Fln was assessed under the conditions of the model oxidative stress. The values of *EC*_50_ of the oxidizers were used in these experiments to imitate oxidative stress conditions. A higher concentration range of Gd@Fln inhibiting the bioluminescence intensity was preliminarily determined and was not used in the experiments.

Both bioluminescent assays, bacterial and enzymatic, were applied to study changes in general toxicity in the oxidizer solutions under addition of Gd@Fln, the antioxidant coefficients *I^rel^_Ox_* were determined as follows:

(3) *I^rel^_Ox_* = *I_Ox+F_*/*I_Ox_*

where *I_Ox_*, *I_Ox+F_* are bioluminescence intensities in oxidizer solutions at *EC*_50_ in the absence and presence of Gd@Fln, respectively, Figure 6.

The bioluminescence enzymatic assay was used to characterize changes in oxidative toxicity in the oxidizer solutions under the fullerenol exposure, the antioxidant coefficients *T^rel^_Ox_* were determined as follows:

*T^rel^_Ox_* = (*T*_0.5_)*_Ox_/(T*_0.5_) *_Ox+F_* (4)

where (*T*_0.5_)*_Ox_* and (*T*_0.5_) *_Ox+F_* are bioluminescence induction periods in the oxidizer solutions in the absence and presence of Gd@Fln, respectively (Figure 6b).

Values of *I^rel^_Ox_* and *T^rel^_Ox_* were determined at different concentrations of Gd@Fln (10^−14^–2∙10^−1^ gL^−1^). Values of *I^rel^_Ox_* > 1 or *T^rel^_Ox_* > 1 revealed a decrease in ‘general’ or ‘oxidative’ toxicities, respectively, under the exposure to Gd@Fln, i.e., antioxidant activity of Gd@Fln in solutions of oxidizers. Values of *I^rel^_Ox_* ≈ 1 or *T^rel^_Ox_* ≈ 1 revealed the absence of the Gd@Fln effects.

All bioluminescence measurements were conducted in five replicates for all solutions. Bioluminescence intensities of bacterial and enzymatic assays were measured without pre-incubation.

### 3.3. Luminol Chemiluminescence Assay

We used luminol chemiluminescence method to evaluate the content of Reactive Oxygen Species (ROS) in the experimental bacterial suspensions and enzymatic solutions [115,116]. This technique is used to determine an integral content of ROS assuming that a dynamic equilibrium of the different ROS forms takes place.

Reagents for the chemiluminescence measurements were: luminol (C_8_H_7_N_3_O_2_) and potassium ferricyanide (K_3_[Fe(CN)_6_]) from Sigma-Aldrich (St. Louis, MO, USA), 3% solution of H_2_O_2_ from Tula Pharmaceutical Factory (Tula, Russia), potassium hydroxide (KOH) from Khimreactiv (Nizhny Novgorod, Russia). All reagents were of chemical grade.

Stock luminol solution (10^−^^2^ M) was prepared as follows: luminol powder was dissolved in 5 mL in 1M solution of KOH and then 5 mL of distilled water was added. The chemiluminescence luminol reaction was initiated by K_3_[Fe(CN)_6_]; maximal value of chemiluminescence intensity was determined. Concentrations of luminol and K_3_[Fe(CN)_6_] in the experimental samples were 2·10^−5^ M and 3·10^−4^ M, respectively. The chemiluminescence registration was carried out immediately following the bioluminescence measurements in the same bacterial and enzymatic samples.

All chemiluminescence measurements were carried out in five replicates.

Initially, the dependences of chemiluminescence intensity on concentration of H_2_O_2_ were determined in distilled water and 3% NaCl solution for enzymatic and bacterial luminescence systems, respectively; they were used as calibration dependences to evaluate ROS content in all experimental samples.

Chemiluminescence intensities were measured in bioluminescence assay systems (bacterial and enzymatic), as well as in bacteria-free/enzyme-free aqueous solutions. Time-courses of *I^rel^* and *ROS^rel^* were obtained at different concentrations of 1,4-benzoquinone (10^−13^–10^−3^ M), Gd@Fln (10^−^^18^–3 gL^−1^), and combinations of 1,4-benzoquinone (at *EC*_50_) and Gd@Fln (10^−^^18^–3 gL^−1^). Optical density of fullerenol or 1,4-benzoquinone solutions was <0.1 at the maximum of the chemiluminescence light emittance (Abs_425_ < 0.1); hence, the effect of “optic filter” was excluded (See Section 3.2).

The relative values of ROS content (*ROS^rel^*) were calculated as ratios of ROS content in the experimental solutions to that in the control solutions.

### 3.4. Equipment

Bioluminescence and chemiluminescence intensity were measured with biochemiluminometer Luminoskan Ascent (Thermo Electron Corporation, Solon, OH, USA) equipped with injector system. All luminescence measurements were carried out at 25 °C. Optical density, *D*, of the fullerenol or 1,4-benzoquinone solutions and bacterial suspensions were measured using a double-beam spectrophotometer UVIKON-943 (KONTRON Instruments, Milano, Italy).

### 3.5. Statistical Processing

The SD-values for *I^rel^, I^rel^_Ox_, T^rel^_Ox_* or *ROS^rel^* were calculated using GraphPad Prism 8 (GraphPad Software, San Diego, CA, USA). They did not exceed 15%, 17%, 13% and 20%, respectively.

To reveal correlations between the bioluminescence signal and ROS concentrations, a statistical dependence between rankings of two variables was analyzed [117], correlation coefficients *r* were calculated.

Statistical processing of the results of bioluminescence and chemiluminescence assays was carried out; *p*-values were calculated with GraphPad Prism 8 using ANOVA. The *p*-values were assessed by Kruskal–Wallis test of two independent sample distributions.

## 4. Conclusions

Our current paper considers the biological activity (toxicity and antioxidant activity) of endohedral gadolinium fullerenol (Gd@Fln) which involved 82 carbon atoms and 40–42 oxygen groups on the surface of the carbon cage. We found that Gd@Fln inhibited bacterial and enzymatic bioluminescence at high concentrations >2·10^−1^ gL^−1^, producing a minimal toxic effect among the previously studied fullerenols. The Gd@Fln moderately activates bacterial cells under lower-concentration exposures: 10^−3^ gL^−1^–2·10^−1^ gL^−1^. The activation processes were accompanied by a consumption of reactive oxygen species (ROS); the bacteria effectively mitigated an increase in ROS content induced by Gd@Fln in aqueous solutions. The results contribute to understanding the molecular mechanism of “hormetic” responses of cells to exposure to low concentrations of bioactive compounds.

The antioxidant activity of Gd@Fln was found at its low and ultralow concentrations (<2·10^−1^ gL^−1^) under the conditions of model oxidative stress, antioxidant coefficients *I^rel^_Ox_* were higher in organic oxidizer solutions than in inorganic ones; this highlights the importance of hydrophobic interactions in redox transformations.

Reactive oxygen species (ROS) were considered as active particles responsible for inhibiting (toxic) and activating effects in the bioassays. We found that both effects are concerned with a decrease in ROS content under the addition of the fullerenol.

We should emphasize that not only excess of ROS can produce a deleterious effect on biological systems, as conventionally stated in biomedical literature, but the lack of ROS can suppress biological functions as well, as is shown in our current investigation.

Hence, our study demonstrated a suitability and high potential for the bioluminescence-based biosensing procedure for the detailed study of the biological activity of carbon nanoparticles with Gd@Fln as an example.

In the frames of our nearest prospective studies, we plan to investigate biological activity of another homologous endohedral fullerenol with lower number of oxygen substituents—Gd@C_82_O_y_(OH)_x_, where x + y = 20–24. We plan to determine its toxic and antioxidant characteristics through similar methods, compare them with those of the Gd@Fln studied in this work, and evaluate its biomedical applicability. According to current theoretical speculations [61], fullerenol with lower number of oxygen substituents should display higher electron affinity, which ensures advanced antioxidant properties.

## Supplementary Materials

The following supporting information can be downloaded at: https://www.mdpi.com/article/10.3390/ijms23095152/s1.

## Abbreviations

EC_50_	effective concentration of oxidizers or fullerenols which inhibited bioluminescence intensity by 50%
F	fullerenol
FMN	flavinmononucleotide
Gd@Fln	Gd@C_82_O_y_(OH)_x_, where x + y = 40–42
I	bioluminescence intensity
IR	infrared
MRI	magnetic resonance imaging
NADH	nicotinamide adenine dinucleotide, disodium salt, reduced
Ox	model oxidizer
ROS	reactive oxygen species
T	bioluminescence induction period
XPS	X-ray photoelectron spectroscopy
