# Supplementary material for: Endohedral Gd-Containing Fullerenol: Toxicity, Antioxidant Activity, and Regulation of Reactive Oxygen Species in Cellular and Enzymatic Systems
Source: Int J Mol Sci. 2022 May 5;23(9):5152. doi: 10.3390/ijms23095152 (PMC9106034; doi:10.3390/ijms23095152)
Supplement: Supplementary file 1 [file ijms-23-05152-s001.zip › ijms-1663685-supplementary 2.pdf]

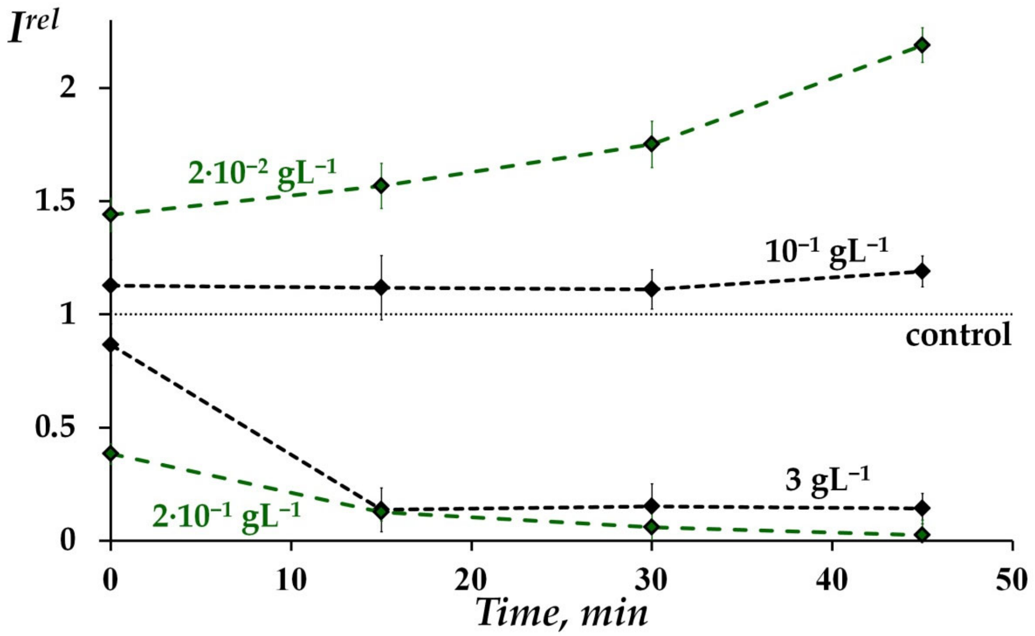

Figure S1. Relative bioluminescence intensity, I^{rel}, vs. time of exposure to fullerenol Gd@Fln of two concentrations in bacterial suspensions (green lines), in enzymatic system (black lines). “Control” corresponds to the absence of Gd@Fln in the experimental solutions.

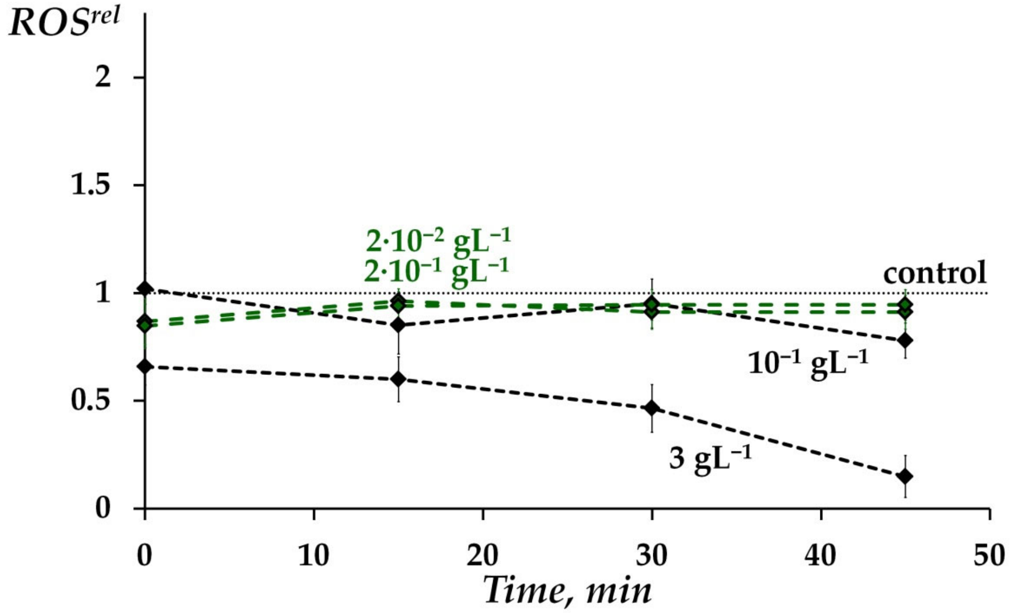

Figure S2. Relative ROS content, ROS^{rel}, vs. time of exposure to fullerenol Gd@Fln of two concentrations in bacterial suspensions (green lines), in enzymatic system (black lines). Concentration of ROS in the control bacterial suspension decayed from $4.8{\cdot}10^{-6}$ M to $4.3{\cdot}10^{-6}$ M, in control enzymatic system increased from $1.9{\cdot}10^{-5}$ M to $4.7{\cdot}10^{-5}$ M for the time of experiment. “Control” corresponds to the absence of Gd@Fln in the experimental solutions.

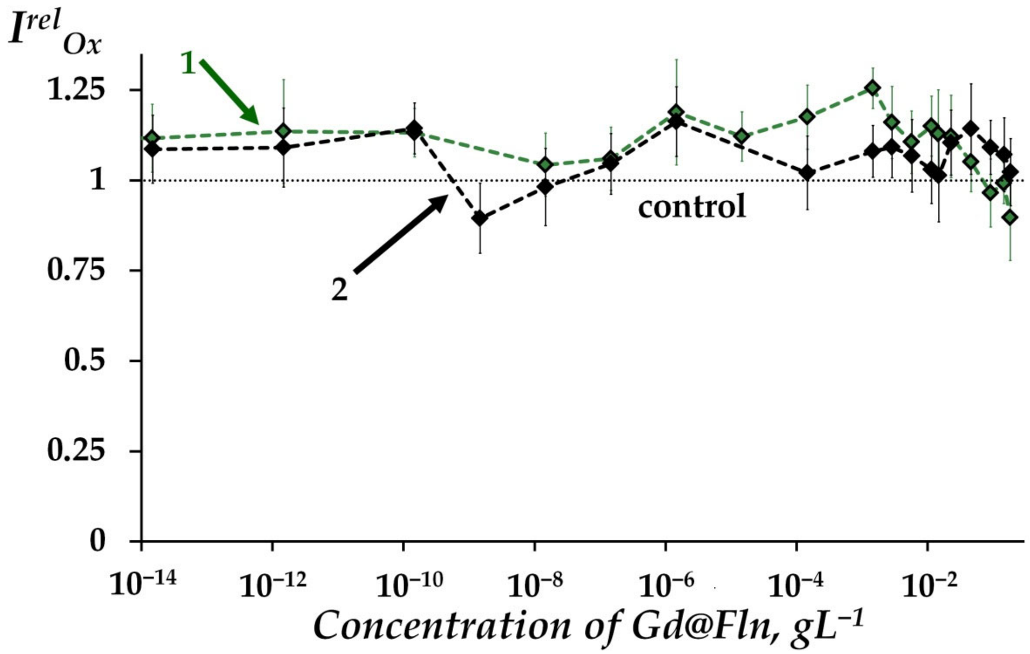

Figure S3. Antioxidant coefficients I^{rel}_{Ox} vs. concentration of fullerenol Gd@Fln in bacterial suspension (1) and enzymatic system (2) in solutions of $K_3[Fe(CN)_6]$ at $EC_{50} = 10^{-3}$ M and $EC_{50} = 10^{-6}$ M, respectively. Time of exposure to Gd@Fln was 1 min. “Control” corresponds to the absence of Gd@Fln in the experimental solutions.

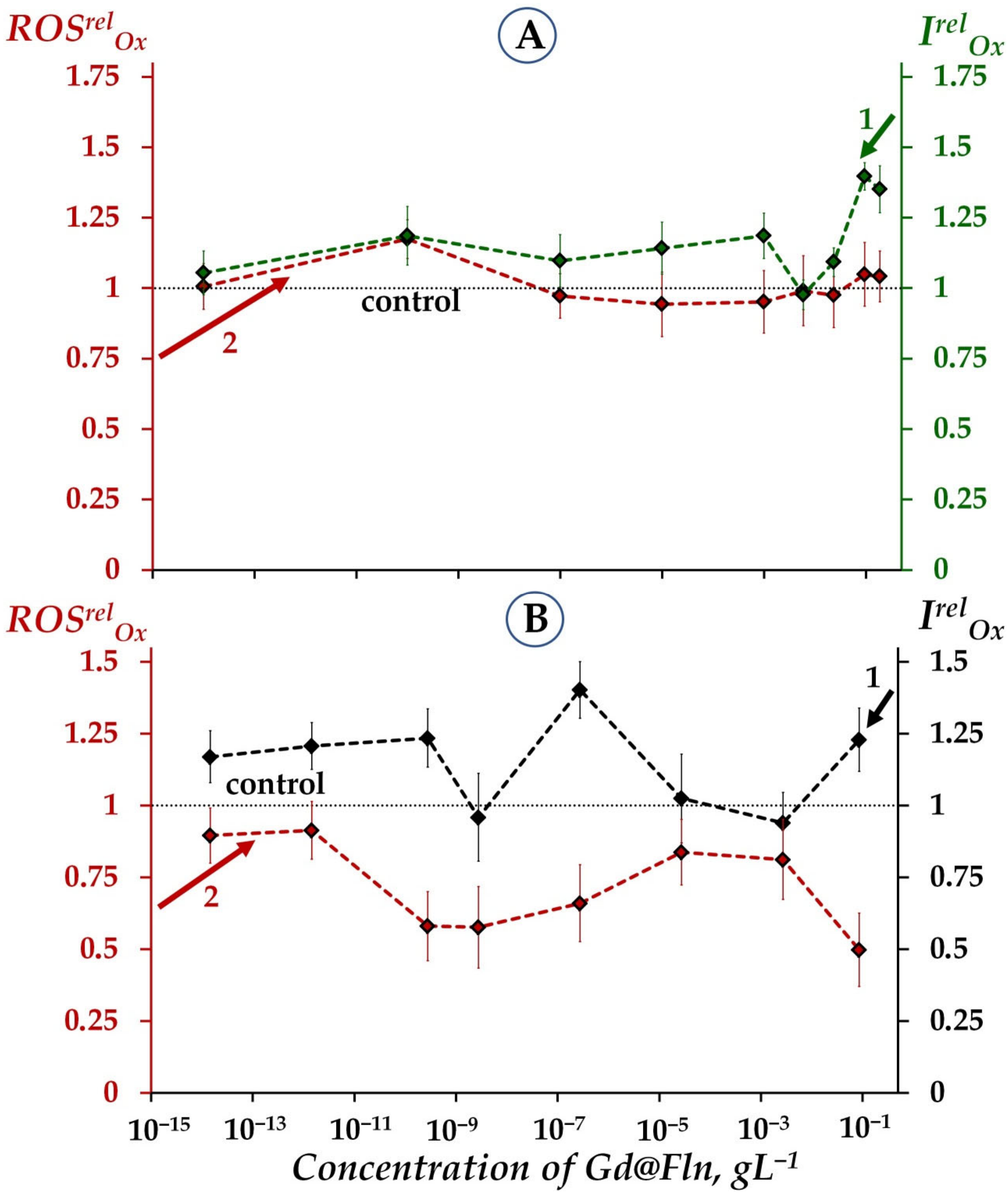

Figure S4. Antioxidant coefficients, I^{rel}_{Ox}, (1) and relative ROS content, ROS^{rel}, (2) in bacterial suspension (A) and enzymatic system (B) vs. concentration of fullerenol Gd@Fln. Time of exposure to Gd@Fln was 5 min. Concentrations of ROS in the control bacterial suspension (bacteria+1,4-benzoquinone at EC_{50} = $8 \cdot 10^{-7}$ M) and enzymatic system (enzymes+1,4-benzoquinone at EC_{50} = 10^{-5} M) were $6.4 \cdot 10^{-6}$ M and $6 \cdot 10^{-5}$ M, respectively. “Control” corresponds to the absence of Gd@Fln in the experimental solutions.

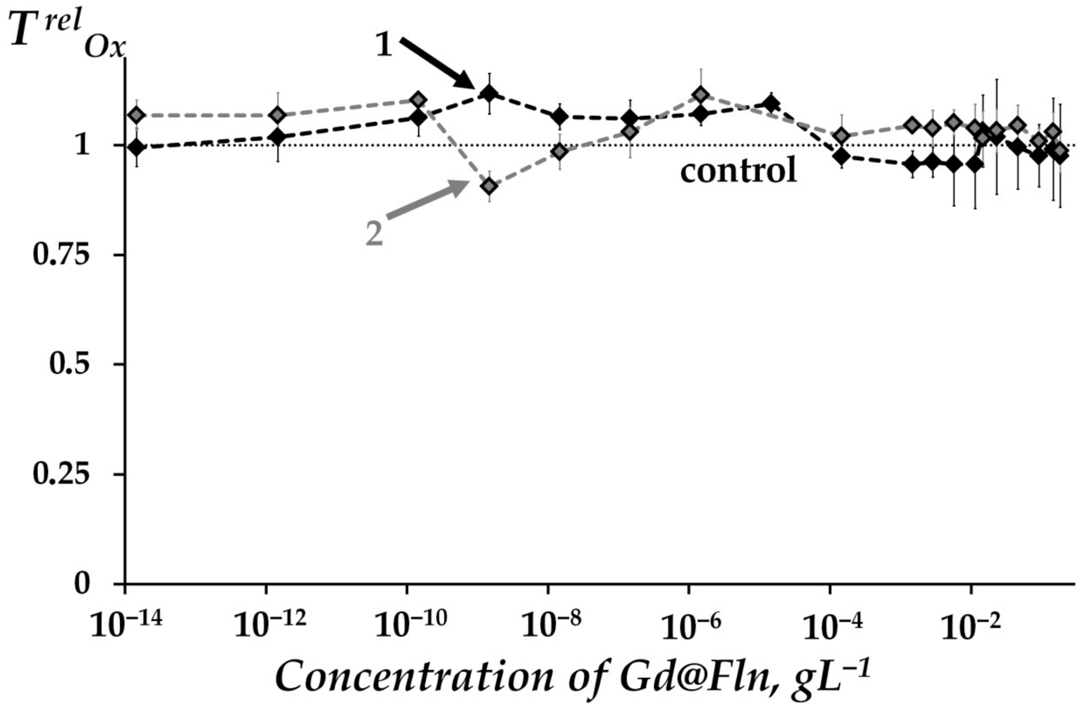

Figure S5. Antioxidant coefficients T^{rel}_{Ox} vs. concentration of fullerenol Gd@Fln in enzymatic system in solutions of 1,4-benzoquinone at *EC50* =10^{-5} M (1) and $K_3[Fe(CN)_6]$ at EC_{50} = 10^{-6} M (2). Time of exposure to Gd@Fln was 1 min. “Control” corresponds to the absence of Gd@Fln in the experimental solution.

Synthesis and characterization of Gd@Fln (Figure S6, S7)

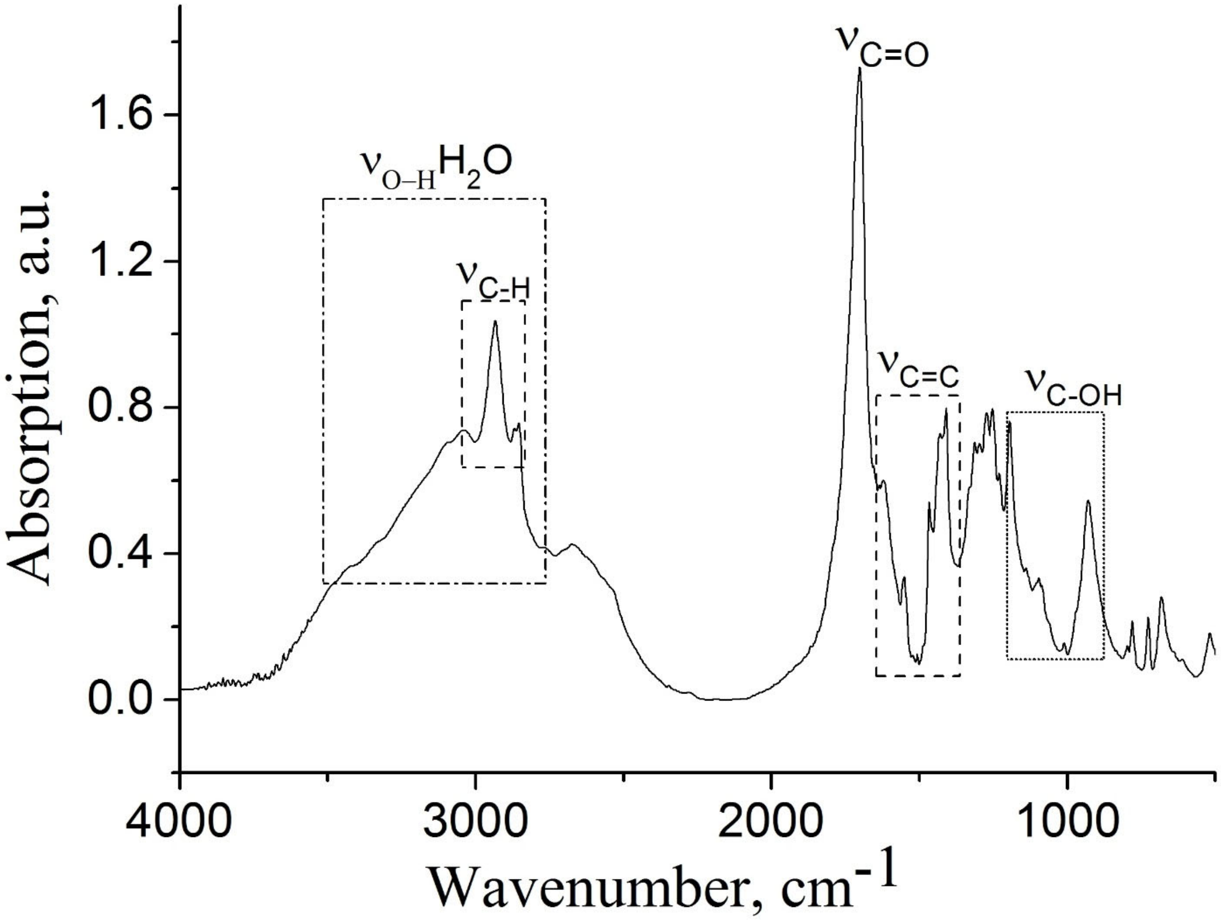

Figure S6. IR spectrum of Gd@Fln.

Synthesis of Gd@Fln was carried out by prolonged endohedral metallofullerene Gd@C_{82} treatment with nitric acid, followed by water washing of intermediate products at 85^{0}C. The presence of -OH groups was confirmed by IR spectroscopy (Fourier-IR spectrometer Vertex 70), Figure S6. Characteristic bands at 3427, 1627, 1390, and 1078 cm^{-1} were registered, showing the presence of oxidized units on the carbon cage. Wave number values 3427 cm^{-1} and 1390 cm^{-1} corresponded to vibrations $^{\nu}$O-H and $^{\delta}$O-H. The bonds of carbon and oxygen were characterized by wave numbers of 1703 cm^{-1} ($^{\nu}$C=O) and 1078 cm^{-1} ($^{\nu}$C-O), which presented carbonyl, ketone, alcohol groups *[Arrais, A.; Gobetto,R.; Rossetti, R.; Diana, E. Synthesis and Spectral Characterization of Water-Soluble Derivatives of C_{70} and High-Order Fullerene Mixture (C_{76}, C_{78} and C_{84}) Achieved by Chemically Induced Air Oxidation. NDFCT.* ***2006****, 16, 79-96].*

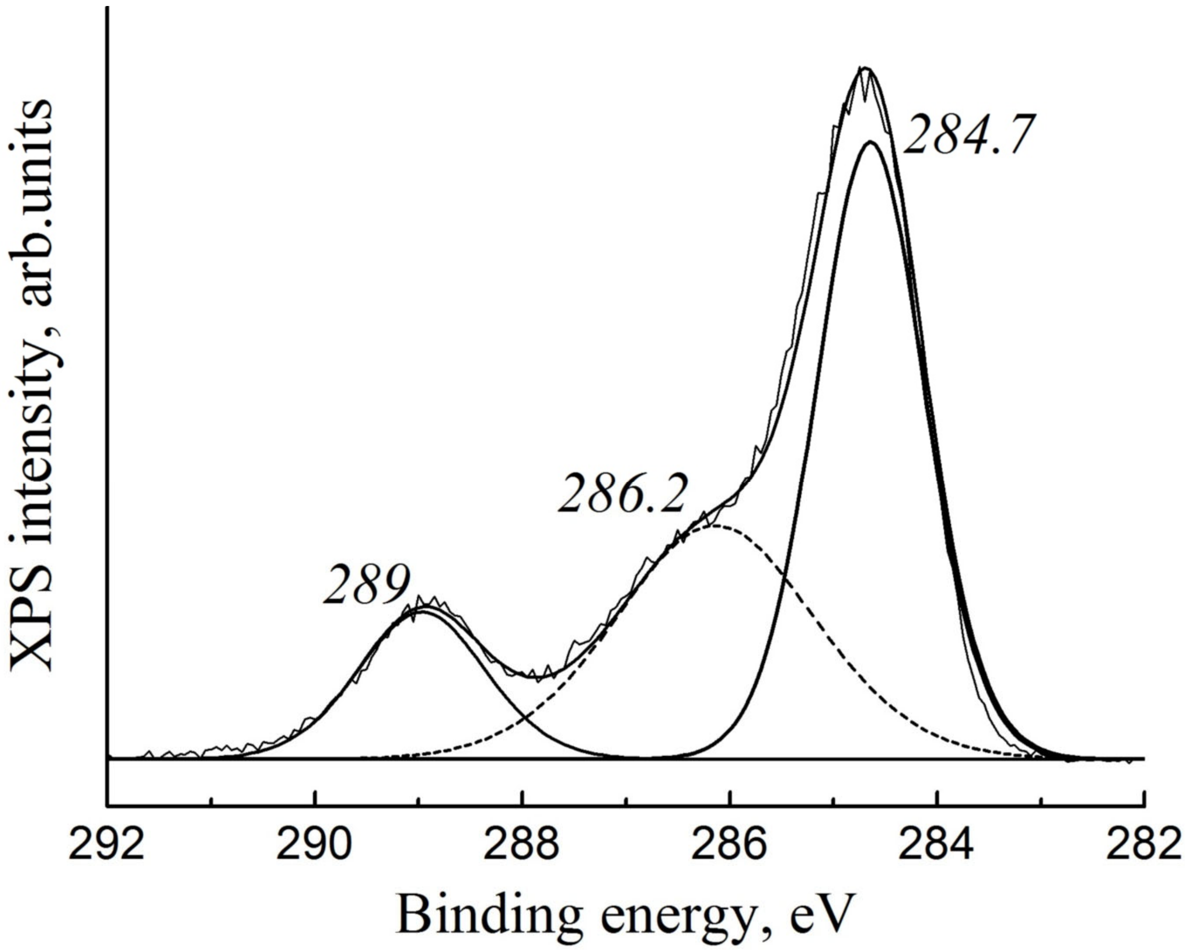

Figure S7. XPS of Gd@Fln (C1s line).

The X-ray photoelectron spectroscopy (UNI-SPECS (SPECS) spectrometer) was used to determine the number of functional groups (Figure S7). The binding energies of 284.8; 286.2; 289 eV, which are assigned to C-C (50.6%), C-O (35.2%) and C=O (14.2%) respectively, were registered in the photoelectron spectrum of the Gaussian/Lorentzian sample; corresponding components were obtained by deconvolution of the C1s line *[Li, J.; Wang, T.; Feng, Y.; Zhang, Y.; Zhen, M.; Shu, C.; Jiang, L.; Wang, Y.; Wang, C. A water-soluble gadolinium metallofullerenol: facile preparation, magnetic properties and magnetic resonance imaging application. Dalton Trans.* ***2016****, 45, 8696-8699. DOI: 10.1039/c6dt00223d; Georgieva, A.T.; Pappu, V.; Krishna, V.; Georgiev, P.G.; Ghiviriga, I.; Indeglia, P.; Xu, X.; Fan, Z.H.; Koopman, B.; Pardalos, P.M.; Moudgil, B. Polyhydroxy fullerenes. J. Nanopart. Res.* ***2013****, 15, 1690. DOI: 10.1007/s11051-013-1690-6]*. The number of functional groups was calculated based on the proportion of carbon atoms chemically bonded to oxygen (49.4% for the test sample), that is, in fullerene, on average, 40.5 carbon atoms out of 82 are bonded to oxygen. It is known that the number of OH groups attached to fullerene must be even *[Wang, B.-C.; Wang, H.W.; Tso H.C.; Chen, T.-L.; Chou, Y.-M. Theoretical studies of $C_{70}(OH)_n$ (n=14, 16, 18 and 20) fullerenols. J. Mol. Struct. (Theochem.)* ***2002****, 581, 177-186. DOI: 10.1016/S0166-1280(01)00756-4]*; the average composition of the product was $Gd@C_{82}O_x(OH)_y$, where x+y=40–42.
